# Harmonizing definitions in liquid biopsy: A terminology framework by the international society of liquid biopsy

**DOI:** 10.1016/j.jlb.2026.100462

**Published:** 2026-03-25

**Authors:** Eloísa Jantus-Lewintre, Silvia Calabuig-Fariñas, David Gandara, Luis Raez, Natasha B. Leighl, Nicola Fusco, Ola Khorshid, Clara Mayo de las Casas, Carolina Reduzzi, Yuksel Urum, Charu Aggarwal, Maria José Serrano, Christian Rolfo, Umberto Malapelle

**Affiliations:** a Instituto Interuniversitario de Investigación de Reconocimiento Molecular y Desarrollo Tecnológico (IDM), Universitat Politècnica de València, Universitat de València, Valencia, Spain; b Unidad Mixta UPV-CIPF de Investigación en Mecanismos de Enfermedades y Nanomedicina, Universitat Politècnica de València, Centro de Investigación Príncipe Felipe, Valencia, Spain; c Molecular Oncology Laboratory, General University Hospital Research Foundation, Valencia, Spain; d Department of Pathology, Universitat de Valéncia, Valencia, Spain; e Centro de Investigación Biomédica en Red Cáncer (CIBERONC), ISCIII, Madrid, Spain; f Deparment of Medicine, University of California Davis Comprehensive Cancer Center, Sacramento, CA, USA; g Thoracic Oncology Program, Memorial Cancer Institute at Memorial Healthcare System, Florida Cancer Center of Excellence, Institute of Human Health and Disease Intervention, Florida Atlantic University (FAU), Miami, Florida, USA; h Division of Medical Oncology and Hematology, Princess Margaret Cancer Centre, Toronto, Canada; i Division of Pathology, European Institute of Oncology IRCCS, Milan, Italy; j Department of Oncology and Hemato-Oncology, University of Milan, Milan, Italy; k Medical Oncology Department NCI Cairo University, Cairo, Egypt; l Hospital Universitari Dexeus. Pangaea Oncology Laboratory, Barcelona, Spain; m Instituto Oncologico Dr Rosell, Barcelona, Spain; n Liquid Biopsy Platform, Department of Medicine, Division of Hematology-Oncology, Weill Cornell Medicine, New York, NY, USA; o Department of Medical Oncology, Ankara University School of Medicine, Ankara, Turkey; p Division of Oncology, Department of Medicine, University of Pennsylvania, Philadelphia, USA; q Department of Pathology, Virgen de las Nieves University Hospital, IBS-Granada, GENYO, Centre for Genomics and Oncological Research, Pfizer/University of Granada/Andalusian Regional Government, Granada, Spain; r Diane Nye and Michael Rayden Chair in Innovative Cancer Research, Division of Medical Oncology, OSUCCC-James, The Arthur G. James Comprehensive Cancer Center, Ohio, USA; s Department of Public Health, University of Naples Federico II, Naples, Italy

**Keywords:** Liquid biopsy, Terminology framework, Harmonized definitions, Circulating tumor DNA (ctDNA), Circulating tumor cells (CTCs), Extracellular vesicles, Tumor fraction, Variant allele frequency, Molecular residual disease (MoRD), Early cancer detection, Precision oncology

## Abstract

The rapid expansion of liquid biopsy (LB) throughout the cancer care continuum is evidenced by the striking increase in annual publications, rising from approximately 100 per year in the early 2000s to more than 2200 in recent years. This exponential growth reflects the accelerating impact of LB across precision medicine. However, the field's evolution has been accompanied by inconsistent use of key terminology, creating barriers to clear communication, comparability of results, and appropriate clinical translation. To address this challenge, the International Society of Liquid Biopsy (ISLB) presents a comprehensive and harmonized terminology framework, an essential and timely effort to support the coherent advancement of the LB discipline.

ISLB defines LB as the analysis of cells, nucleic acids, proteins, metabolites, and extracellular vesicles used to interrogate pathological or specific physiological conditions, obtained from bodily fluids, primarily through minimally invasive methods. Here we discuss relevant biospecimens, emphasizing their distinct sources and molecular characteristics.

A central component of the manuscript is a rigorous clarification of analyte-specific terminology. This includes circulating free DNA and RNA (cfDNA, cfRNA), circulating tumor DNA and RNA (ctDNA, ctRNA), tumor fraction (TF), variant allele frequency (VAF), circulating tumor cells (CTCs), disseminated tumor cells (DTCs), circulating tumor microemboli (CTMs), extracellular vesicles (EVs), tumor-educated platelets (TEPs), soluble proteins, and metabolomic signatures. The manuscript also outlines the analytical methodologies, that enable sensitive detection of low-abundance tumor-derived signals.

Clinical applications of LB in Oncology are defined across the disease continuum, including early cancer detection, molecular residual disease (MoRD) assessment, predictive biomarker evaluation, and tumor monitoring.

By establishing a cohesive vocabulary, ISLB provides a robust reference framework that will evolve with scientific progress and guide the integration of LB into precision medicine.

## 1 Introduction

**Fig. 1:**
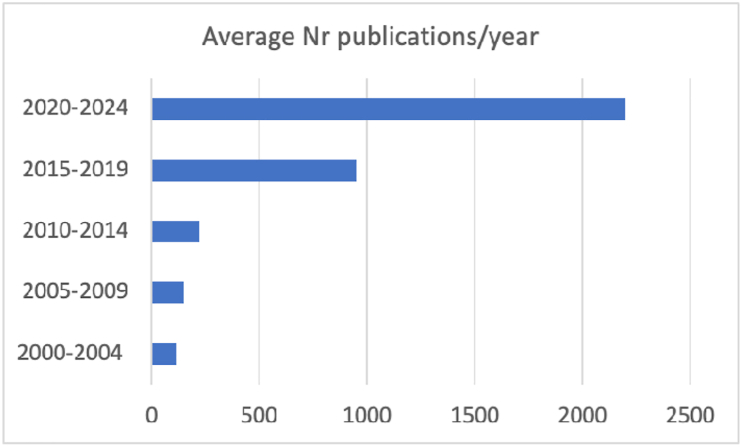
Average number of publications per year found under the topic “liquid biopsy” in the Web of Science from 2000 to 2024.

The field of liquid biopsy (LB) has experienced a remarkable surge in scientific interest and research activity over the past two decades, as reflected by the increasing number of publications indexed in the Web of Science. A search using the term *“liquid biopsy”* reveals a steady rise in the average number of publications per year—from 112 in the early 2000s (2000–2004) to over 2200 annually between 2020 and 2024 (Fig. 1). This dramatic growth highlights the expanding recognition of LB as a transformative tool in precision medicine, driven by technological advancements and a broadening range of clinical applications.

**Fig. 2:**
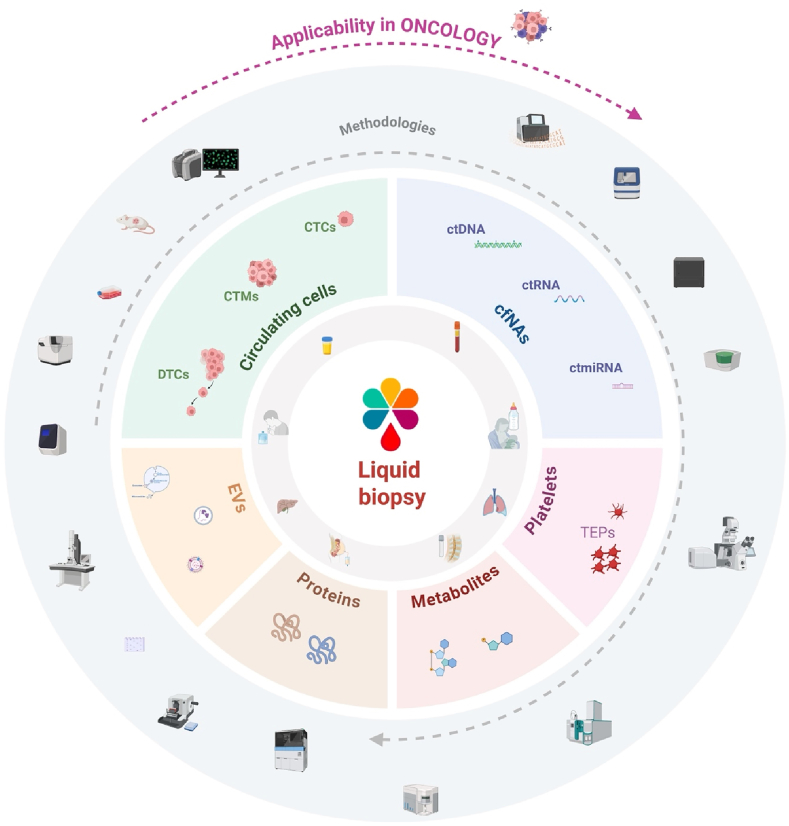
Key definitions in Liquid Biopsy: Analytes, Technologies, and Clinical Applications. The figure is organized into four concentric layers. **The inner circle** shows different types of biological fluids that can be included in the concept of liquid biopsy. **The second layer** depicts core concept of liquid biopsy, representing the full spectrum of circulating analytes accessible and links each class to the methodological platforms used for their isolation and/or analysis. **The outer layer** corresponds to the variety of methodological approaches that can be used for the analysis of liquid biopsy. Abbreviations: Circulating free nucleic acids (cFNAs), circulating tumor DNA (ctDNA), circulating tumor RNA (ctRNA), circulating tumor micro RNA(ctmiRNA), tumor-educated platelets (TEPs), extracellular vesicles (EVs), circulating tumor cells (CTCs), circulating tumor microemboli (CTMs), disseminated tumor cells (DTCs). Image created in https://BioRender.com.

The International Society of Liquid Biopsy (ISLB) (www.islb.info) was founded in 2017 with a clear objective: to become the leading scientific reference in the field of LB to provide education worldwide and to serve as a unifying platform for all stakeholders. Central to this mission is the need to harmonize and clarify key definitions, establishing a shared framework that supports consistent communication among researchers, clinicians, and technologists. To address this critical gap, we provide an overview of essential LB-related terminology aimed at standardizing concepts and to consolidate a coherent conceptual structure in this rapidly evolving field. A graphical abstract of this article is summarized in Fig. 2.

### 1.1 Liquid biopsy

LB, as defined by ISLB, involves the analysis of various components such as cells, nucleic acids, proteins, metabolites, extracellular vesicles, associated with pathological or specific physiological conditions and obtained from several bodily fluids collected primarily through minimally invasive methods. This approach enables the detection of clinically relevant biomarkers in LB offering dynamic real-time diagnostic, prognostic, and predictive insights, ultimately contributing to improved clinical outcomes.

Nowadays, LB is widely implemented in oncology, to complement tissue-based molecular analysis when scant material impact on the success of molecular analysis in advanced stage cancer patients. ISLB has expanded the definition of LB reflecting the current and evolving use of the term, applying LB beyond oncology to encompass a broader spectrum of pathophysiological states and aligning more closely with current clinical and research practices.

In this scenario, harmonized handling procedures spanning from pre-analytical management of diagnostic samples to guidelines for reporting molecular findings are crucial to optimize clinical stratification of patients.

### 1.2 Bodily fluids

LB analysis can be technically performed on a wide series of bodily fluids, each offering unique advantages depending on the anatomical location of the biological process under investigation. In this section, we present an overview of the most useful LB biospecimens.

**Blood**: The most commonly biological source to isolate analytes, peripheral blood, contains different matrixes of analysis like cells, extracellular vesicles and biomolecules (DNA, RNA, proteins and metabolites). Many of these analytes are enriched and more stably assessed in the plasma fraction. Proper selection of blood collection tubes and standardized preanalytical workflows are essential to preserve specific components that can influence downstream applications. Consequently, plasma is the preferred and most widely used fraction of whole blood for biomarker analysis in LB.

**Cerebrospinal fluid** (CSF): is a valuable biospecimen for diagnosing and monitoring neurological conditions, including brain tumors. It contains nucleic acids, proteins, and metabolites arising from tissues in the brain and spinal cord. Particularly, the blood brain barrier blocks blood diffusion of circulating analytes between CSF and blood and vice versa. On this basis, CSF is considered an abundant source of analytes for patients with neurological diseases [1,2]. Despite these advantages, CSF collection is impacted by the invasive nature of its collection, decreasing its clinical implementation.

**Urine**: is an ideal source of biomarkers for the detection of kidney, bladder, and prostate diseases, largely due to the anatomical proximity of these organs to the urinary tract. This close anatomical relationship facilitates the direct release of disease-related molecules, such as nucleic acids, proteins, and metabolites, into the urine[3,4]. Beyond urogenital diseases, urine is also used in prenatal testing, metabolic disorder screening, cancer biomarker identification, and infectious disease diagnostics. As peripheral blood, urine collection is safe and does not require technically complex handling procedures.

**Saliva**: Emerging as a LB medium for its role in detecting oral, oropharyngeal and even systemic diseases. As a readily accessible biofluid, it can reflect physiological and pathological changes in the body, coming into direct contact with oral lesions, making it an ideal choice for non-invasive screening. Several trials demonstrated high concordance rate between saliva and peripheral blood in detecting tumour derived analytes, thereby paving the way for further investigation of saliva-based LB approaches [[5], [6], [7], [8]].

**Pleural effusion** (PE): This fluid, which accumulates in the pleural cavity due to infections, cancer, or inflammatory conditions, provides useful information in diagnosing lung diseases, mesothelioma or infections like tuberculosis. Interestingly, PE may significantly shift the clinical paradigm for tumor patients because both cytological and LB samples may derive from unique sampling collection [9].

**Sputum**: is a mixture of saliva, mucus, and phlegm originating from the upper respiratory tract. It can be collected naturally through coughing or induced through stimulation [10]. This respiratory secretion has been analyzed for genetic and epigenetic changes, aiding in the detection of lung cancer at different stages. Additionally, sputum testing has been explored in chronic smokers without cancer, who face an elevated risk of developing lung malignancies, as well as for diagnosing other non-malignant respiratory conditions. Further investigations are needed to identify molecular signatures able to distinguish between healthy and tumor patients.

**Bile**: A digestive fluid produced by the liver, stored in the gallbladder, and released into the duodenum, it has a complex composition that undergoes significant changes under various pathological conditions. Notably, studies have demonstrated that detecting mutations in bile-derived cell-free DNA (cfDNA) can surpass blood-based analysis in the early diagnosis of biliopancreatic tumors [11,12]. As a valuable source of biomarkers, bile contains a diverse range of analytes, including lipids, metabolites, proteins, and nucleic acids, making it a promising fluid for LB applications.

**Ascitic fluid:** consisting in the pathological accumulation of serous fluid in the peritoneal cavity, the space between the two layers of the peritoneum that lines the abdominal organs. In physiological conditions contains less than 50 ml, serving primarily as a lubricant. However, when the delicate balance between production and absorption is disrupted, ascites can develop. In such cases, the fluid volume may range from few hundred ml to several liters. While traditionally used for diagnostic cytology, microbiology, and biochemical profiling, ascitic fluid is increasingly recognized as a rich, informative biofluid [9]. Rather than being a passive transudate or exudate, it reflects the molecular, cellular, and proteomic environment of the peritoneal cavity**.** It can simultaneously capture tumor cells and tumor-derived analytes in malignancies with peritoneal dissemination (e.g., ovarian, colorectal, or pancreatic cancer), thereby enhancing diagnostic accuracy in this clinical setting.

**Maternal milk:** a complex biofluid secreted by the mammary glands during lactation, primarily composed of water, lipids, proteins, carbohydrates, immune cells, and bioactive molecules. While its physiological role is to nourish and immunologically protect the newborn, maternal milk also reflects the metabolic and cellular state of the mammary gland. Under both physiological and pathological conditions, it contains a range of analytes, including cfDNA, extracellular vesicles, RNA species, and proteins, that are increasingly recognized as valuable biomarkers. Although traditionally studied in the context of infant health and nutrition, recent research has demonstrated the utility of maternal milk in detecting tumor-derived analytes in lactating women, particularly in the early detection and molecular profiling of breast cancer [13]. Far from being a passive secretion, maternal milk mirrors the local and systemic biological processes occurring within the lactating breast.

### 1.3 Liquid biopsy in oncology

In the era of precision medicine, tumor tissue still remains the gold standard for cancer molecular profiling in most clinical settings. In particular, diagnosis of advanced-stage cancer is frequently based on fine needle aspiration (FNA), which often results in a sample adequate for histopathological studies but insufficient for comprehensive molecular analysis. Here, LB represents a minimally invasive approach for comprehensive genomic profiling. Moreover, it is particularly valuable in clinical scenarios requiring serial sampling, such as the identification of mechanism of acquired resistance to targeted therapies and monitoring disease response to therapy. It has been demonstrated that LB can also provide critical insights into assessing relapse risk after treatment and may serve as a fundamental component of future cancer screening protocols [14].

LB encompasses various analytes, including circulating tumor cells (CTCs) and macromolecular tumor products such as circulating tumor DNA (ctDNA), circulating tumor RNA (ctRNA), extracellular vesicles (EVs), tumor-educated platelets (TEPs), tumor proteins, metabolites, among others. These biomarkers offer valuable information about the cells from which they originate, reflecting diverse biological processes involved in tumorigenesis and progression [15].

#### 1.3.1 Concepts

##### 1.3.1.1 Circulating cell-free nucleic acids

Circulating cell-free nucleic acids (cfNAs) are extracellular fragments of nucleic acids released by cells into bodily fluids through passive processes such as apoptosis, necrosis, netosis and also by active secretion mechanisms. These nucleic acids can originate from both healthy and diseased cells, including tumor cells, making them valuable for analysis of biomarkers in different clinical settings.

Typically, cfDNA consists of double-stranded fragments derived from both nuclear and mitochondrial DNA. In healthy individuals, cfDNA primarily originates from hematopoietic cells and exhibits a characteristic fragment size of approximately 160 base pairs (bp), corresponding to the length of DNA wrapped around a nucleosome plus the linker DNA. Notably, the fragmentation pattern of cfDNA differs between healthy individuals and cancer patients, paving the way for the use of fragmentomic profiles as a promising tool for early cancer detection, as discussed below.

##### 1.3.1.2 Circulating tumor nucleic acids

In oncology, other frequently used terms include circulating tumor nucleic acids (ctNA), which comprise DNA (ctDNA) and RNA (ctRNA), including different RNA species such as messenger RNAs (mRNA), microRNAs (miRNA), long non-coding RNAs (lncRNA), circular RNAs (circRNA), among others.

##### 1.3.1.3 Circulating tumor DNA (ctDNA)

ctDNA refers to short fragments of DNA that are shed into the bloodstream by cancer cells as a result of cellular processes such as apoptosis, necrosis, or active secretion. ctDNA represents a tumor-specific small subset of cfDNA fragments harboring specific genomic and epigenetic modifications.

Given the low abundance of ctDNA in peripheral blood, highly sensitive technologies for the reliable detection of tumor-specific molecular alterations. Moreover, ctDNA is characterized by a short half-life (approximately 15 min), which requires optimized pre-analytical procedures, such as the use of specialized blood collection tubes and careful plasma processing, to preserve sample integrity for molecular analysis.

Notably, ctDNA fragments are typically shorter than non-tumor cfDNA, with a median size of approximately 134–144 base pairs. Based on this observation, novel technical approaches are being developed to characterize specific fragmentation patterns in early-stage cancer patients, thereby improving the clinical sensitivity and specificity of current diagnostic methods.

**Fig. 3:**
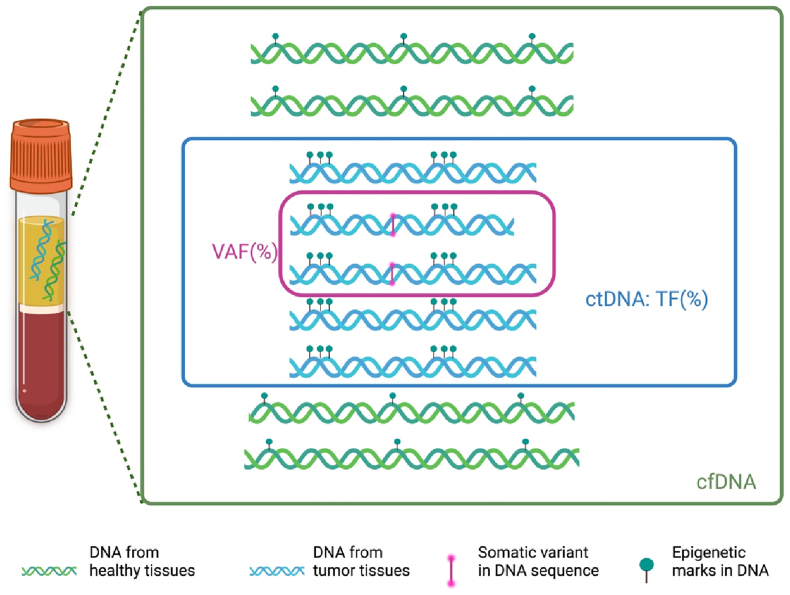
Graphical description of the concepts of Tumor Fraction (TF) and Variant Allele Frequency (VAF). Image created in https://BioRender.com.

Tumor fraction: ctDNA typically comprises only a small portion of the total cfDNA present in the bloodstream. So, the term Tumor Fraction (TF) refers to the percentage of ctDNA within a given cfDNA sample serving as a key parameter to depict the burden of tumor-derived genetic material in bloodstream (Fig. 3). TF is technically calculated leveraging advanced bioinformatic tools able that analyze several cfDNA features: i) aneuploidy based approach, ii) identification of tumor specific epigenetic marks or iii) measuring allele frequencies of tumor derived genomic hallmarks, like somatic variants and genomic rearrangements [16,17].

Shedder and Non-shedder cancers: TF is widely influenced by several biological factors, such as histology, location, vascularity, necrosis, proliferative capacity, grade, stage and tumor burden. These factors affect the amount of ctDNA shed into the bloodstream, leading to a classification of tumors as either shedders (those that release detectable ctDNA) or non-shedders (those that release very low levels of ctDNA that fall below the analytical limit of detection)[18]. This characteristic explains why plasma ctDNA assays may yield negative results and fail to provide informative molecular data for biomarker assessment in non-shedder tumors.

Variant allele frequency (VAF): Is a technical parameter to measure the proportion of DNA molecules that carry a specific variants, relative to the total number of DNA molecules mapped to the same genomic position (including both wild-type and variant alleles) (Fig. 3). In some situations, the term Mutant Allele Frequency (MAF) may be used to refer to the same concept. In the context of LB in Oncology, VAF provides insights into tumor clonality, indicating how abundant a particular somatic variant is present in cfDNA. VAF provides insights into the tumor's genomic landscape, mutation load and its evolution over time. It serves as an important metric for targeting dominant cancer cell populations, detecting minimal residual disease (MRD), and monitoring response or resistance to treatment. In general, most of the solutions used for ctDNA analysis are equipped with bioinformatic pipelines that automatically report variant allele frequency (VAF). However, the detection of low-frequency variants (<1.0%) with clinical relevance often requires advanced, integrative bioinformatic approaches to accurately identify trace amounts of ctDNA in the sample.

**Types of analysis in ctDNA:** NGS, dPCR, methylation, fragmentomics, genomic scars (HRD).

Low ctDNA abundance in bloodstream dramatically impacts on the analytical approaches diagnostically available to detect clinically informative molecular alterations. Overall, highly sensitive technologies are recommended to successfully identify low traces of ctDNA mutations in diagnostic samples.

Next Generation Sequencing (NGS): NGS enables the simultaneous detection of a broad range of genomic alterations—including point mutations, insertions/deletions, copy number variations (CNV), and gene rearrangements—across multiple genomic regions. Plasma NGS can also reveal genomic heterogeneity in cancer, evaluating the differential contribution of metastatic sites rather than tissue biopsy of a single site. However, its effectiveness can be limited by the low abundance of ctDNA in plasma and the presence of background noise from non-tumor-derived DNA [19,20]. In this scenario, ultradeep NGS systems built to selectively capture/amplify mutant alleles have demonstrated greater technical sensitivity than conventional NGS assay. Particularly, these strategies may be found in MRD-directed assays or in early stage diagnosis. Unfortunately, the implementation of NGS platforms in routine diagnostic practice requires highly trained personnel and specialized infrastructure. To address this limitation, singleplex technologies have been developed as complementary tools that can be more easily integrated into diagnostic workflows alongside NGS platforms. Most recently, “multi-omic” platforms incorporating DNA fragmentomics, methylation or proteomics have been demonstrated to be effective additions to standard NGS testing, as discussed below.

Digital PCR (dPCR): is a highly sensitive molecular technique able to absolute quantify specific NA sequences. These systems are based on partitioning systems of targeted DNA samples into thousands of individual reactions (both in emulsion and in plate), each ideally containing zero or one target molecule. After amplification, each partition yields a binary output—positive or negative—which is then analyzed using Poisson statistics to calculate the precise concentration of the target sequence. This partition-based approach allows for accurate detection of low-frequency variants, with a MAF% detection limit as low as 0.01%. As a result, dPCR is particularly well-suited for the highly sensitive identification of rare mutations, as well as for treatment monitoring and the detection of MRD [21]. In recent years, this approach has also been adapted to track targeted epigenetic modifications, further expanding its utility in precision oncology.

Methylomic analysis: is based on the comprehensive investigation of DNA methylation patterns within ctDNA fragments in body fluids. DNA methylation, an epigenetic modification involving the addition of methyl groups to cytosine residues in CpG dinucleotides, plays a crucial role in regulating gene expression usually deregulated in cancer. By analyzing these methylation signatures in LB samples, methylomic profiling enables the identification of tumor-specific epigenetic changes with high specificity and sensitivity. Unlike mutation-based analyses, methylomics capture cancer-associated epigenetic alterations that often occur early in tumorigenesis and may be tissue- or cancer-type specific [22,23]. On this basis, several trials were built to discover tumor specific methylation patterns able to specifically identify cancer origin.

Fragmentomics: refers to the genome-wide analysis of the physical characteristics of ctDNA fragments, such as their size distribution, end motifs, genomic location, and nucleosomal patterns, to extract biologically and clinically relevant information. Fragmentomic approaches do not rely on the detection of specific genetic alterations but instead examine the structural features of DNA fragments that reflect their origin and mode of release cells. In cancer patients, ctDNA tends to show distinct fragmentation profiles compared to non-tumor-derived cfDNA, including shorter average fragment lengths, aberrant cleavage patterns, and altered nucleosome footprints. These features can be leveraged for early cancer detection, tissue-of-origin prediction, and monitoring of tumor dynamics [[24], [25], [26]].

Genomic Scars: refer to persistent patterns of chromosomal aberrations that accumulate in tumor DNA as a consequence of defective homologous recombination (HRD) repair mechanisms, which are critical for the accurate repair of DNA double-strand breaks. In cancers with HRD, such as those harboring *BRCA1/2* mutations, these aberrations lead to characteristic genome-wide alterations, including loss of heterozygosity (LOH), large-scale state transitions (LSTs), and telomeric allelic imbalance (TAI). These structural signatures, collectively termed “genomic scars,” can be detected not only in tumor tissue but also in ctDNA using NGS and computational algorithms[27].

##### 1.3.1.4 Circulating tumor RNA (ctRNA)

ctRNA defines a tumor-specific subset of cfRNA that originates from malignant cells. It can be detected in plasma or other fluids either freely, in association with protein complexes or encapsulated within extracellular vesicles. ctRNA can reflect intra-tumoral dynamic processes on the cellular and intercellular levels.

ctRNA is a source that enables the detection of gene fusions, particularly in cancers where chromosomal translocations generate chimeric transcripts. Historically, ctDNA-based assays often required a high tumor burden to reliably detect structural rearrangements. Although technological advances have substantially improved the sensitivity of ctDNA assays, ctRNA enables the direct detection of fusion transcripts, thereby increasing both sensitivity and specificity for translocation identification. However, ctRNA is inherently less stable than ctDNA, with an estimated half-life shorter than 15 min, which limits its diagnostic applicability for the detection of aberrant transcripts. Consequently, preanalytical handling procedures including sample collection, transportation, and storage, must be carefully optimized to ensure successful ctRNA analysis.

In LB applications, miRNAs, a class of small non-coding RNAs have emerged as promising noninvasive biomarkers in oncology due to their stability in blood and their critical role in post-transcriptional gene regulation. These small RNA molecules are often dysregulated in cancer and can reflect tumor-specific biological processes such as proliferation, apoptosis, invasion, and drug resistance.

##### 1.3.1.5 Circulating soluble proteins

These LB biomarkers are comprised of circulating tumor-associated proteins, released by cancer cells directly or through tumor-induced systemic responses. In this definition we include tumor antigens, enzymes, cytokines, chemokines and soluble mediators measured in plasma or serum in the case of blood samples or in other biological fluids [28].

Circulating protein markers are the most established diagnostic tools for cancer. Common examples include prostate-specific antigen (PSA), cancer antigen 15-3 (CA 15-3), carbohydrate antigen 19-9 (CA 19-9), carcinoembryonic antigen (CEA), and alpha-fetoprotein (AFP), all of which are used for the detection of various tumor types. Despite their clinical use, individual protein markers often suffer from limited sensitivity and specificity. Recently, combining multiple protein markers into composite panels has shown promise in enhancing screening and diagnostic utility, primarily by minimizing false-negative and false-positive results and also by improving predictive information.

##### 1.3.1.6 Metabolomics in biofluids

This definition encompasses the comprehensive analysis of small-molecules metabolites in bodily fluids. These metabolites are the end products of cellular processes and reflect both genetic and environmental influences, making metabolomics a powerful research approach for identifying functional alterations associated with cancer and other pathological conditions.

##### 1.3.1.7 Circulating tumor cells (CTCs)

CTCs are tumor cells that detach from primary or metastatic tumors and spread via blood and/or lymphatic vessels. The phenotype of CTCs can be different and so may present differential levels of potential aggressiveness [29]. Their detection and analysis aid in tracking tumor progression and metastasis.

##### 1.3.1.8 Disseminated tumor cells (DTC)

DTC are cancer cells that have detached from the primary tumor and migrated to distant tissues, most commonly the bone marrow, where they may persist in a dormant or non-proliferative state. Unlike CTCs, which travel transiently through the bloodstream or lymphatic system, DTCs have extravasated from the circulation and taken residence in distant microenvironments. DTCs typically exhibit biological characteristics distinct from CTCs, such as dormancy-associated gene expression profiles and interactions with the local stromal and immune milieu. Their ability to remain undetected and resistant to conventional therapies poses a significant risk for future metastatic relapse, sometimes years after apparent remission [30], [31]. While CTCs reflect ongoing tumor cell dissemination and are primarily used for real-time monitoring of disease activity, DTCs are considered indicators of MRD and long-term metastatic potential. The detection and molecular characterization of DTCs, particularly in the bone marrow, provide critical prognostic information and may help guide adjuvant therapy decisions in the future, especially in breast and prostate cancers [32].

##### 1.3.1.9 Circulating tumor microemboli (CTM)

CTMs are multicellular aggregates of tumor cells, often accompanied by stromal or immune cells, which detach from the primary or metastatic tumor and enter the bloodstream as clusters. Unlike individual CTCs, CTMs retain partial cell–cell adhesion and often exhibit collective migration properties, which are associated with enhanced survival in circulation and increased metastatic potential [33].

CTMs differ biologically and functionally from single CTCs. While single CTCs are generally more vulnerable to shear stress and immune clearance in the bloodstream, CTMs demonstrate higher resistance to apoptosis, a greater likelihood of extravasation, and an increased ability to colonize distant organs. Their clustered nature also enables intercellular signaling and cooperation, contributing to therapy resistance and metastatic efficiency. Studies have shown that the presence of CTMs in blood samples correlates more strongly with poor prognosis and advanced disease stage than the presence of individual CTCs alone [34].

The detection and molecular characterization of CTMs provide valuable insights into tumor heterogeneity, metastatic dynamics, and potential therapeutic targets, highlighting their emerging importance as biomarkers in LB.

##### 1.3.1.10 Circulating genetically abnormal cells (CGACs)

CGACs are rare circulating cells characterized by the presence of somatic genetic or chromosomal abnormalities associated with cancer, encompassing but not limited to CTCs. Defined primarily by genetic alterations rather than phenotypic markers, CGACs preserve cellular integrity and enable single-cell genomic and phenotypic analyses, providing complementary information to cell-free biomarkers in LB applications [35].

##### 1.3.1.11 Tumor-educated platelets and platelet-educated tumor cells

Tumor-educated platelets (TEPs) are blood platelets that have been functionally altered by interactions with cancer cells. These changes occur through the active uptake of tumor-derived EVs, RNA and other biomolecules. As a result, TEPs undergo transcriptomic modifications, making them reflective of the tumor's presence and biological behavior [36]. Due to their dynamic interaction with cancer cells, TEPs carry tumor-specific RNA signatures, which can be identified through mRNA sequencing. These features position TEPs as promising biomarkers in LB for cancer detection, tumor type classification, and even localization of the primary tumor.

On the other hand, the concept of Platelet-Educated Tumor cells (PETs) refers to tumor cells whose behavior is modulated or reprogrammed by interactions with platelets [37]. This process is essentially the inverse of TEPs. In this context, platelets bind to CTCs and facilitate several pro-metastatic functions, such as shielding CTCs from immune surveillance or inducing epithelial to mesenchymal transition in tumor cells increasing their invasiveness and metastatic potential.

##### 1.3.1.12 Extracellular vesicles

Extracellular vesicles (EVs) are membrane-enclosed structures released by cells through active or passive processes, including regulated secretion or as a result of cell death. The International Society of Liquid Biopsy (ISLB), in alignment with the International Society for Extracellular Vesicles (ISEV), endorses the use of the term EV for particles naturally released from cells, characterized by a lipid bilayer and the absence of a functional nucleus, making them non-replicative [38]. These structures contain substantial amounts of biologically active information (DNA, RNAs, miRNAs, proteins, lipids) derived from their cells of origin, which can be transported to other cells or organs under both homeostatic and disease conditions. EVs encompass an heterogeneous populations that can be categorized based on their size, morphological features, cellular origin, lipid profiles, and molecular content or surface markers. The best-known EV subtypes include apoptotic bodies (800–5000 nm), microvesicles (100–1000 nm), and exosomes (40–160 nm)[39]. Based on their distinct biogenesis pathways, EVs comprise exosomes, produced via the endosomal pathway through inward budding of multivesicular body membranes and released following fusion with the plasma membrane, and ectosomes, which arise from direct budding of the plasma membrane [40].

Despite challenging procedures to isolate EVs, these particles are very insightful integrating genomic data on the molecular assessment of tumor patients.

#### 1.3.2 Key terminology in clinical applications of LB in oncology

##### 1.3.2.1 Screening programs

In the context of cancer screening, the term LB can be misinterpreted as indicative of a definitive cancer diagnosis. To mitigate this potential confusion, BLOODPAC recommends using more precise terminology, such as single-cancer detection test (SCD) or multi-cancer detection test (MCD). These terms explicitly reflect the screening intent of these assays and distinguish them from diagnostic procedures, thereby facilitating clearer communication among clinicians, regulators, and patients. This distinction is consistent with the recommendations outlined by Clarke et al. and is also supported by ISLB [41].

##### 1.3.2.2 Early cancer detection

This clinical application of LB refers to the identification of cancer at initial, asymptomatic stages before any radiologically detectable disease, through the analysis of tumor-derived material present in body fluids. On this basis, several tumor informed molecules may be identified in depth to early detect tumor patients. Early detection via LB has the potential to significantly improve clinical outcomes by enabling timely therapeutic intervention and increasing the likelihood of curative treatment. Compared to conventional early detection tools, LB offers advantages in terms of safety, repeatability, and comprehensive molecular characterization. Advanced technologies such as ultra-deep NGS, dPCR, and fragmentomic or methylomic profiling are central to enhancing the sensitivity and specificity of LB-based early detection assays. The most promising strategies are based on integrative LB analyses, combining multi-layer omics data, which have demonstrated increasing accuracy in patient stratification. However, these approaches still require further validation before they can be implemented in routine clinical practice [19,23].

##### 1.3.2.3 Residual disease assessment by LB

The term Minimal Residual Disease (MRD), also referred to as measurable residual disease, was originally developed in the context of hematologic malignancies, where it describes the persistence of residual malignant cells after treatment despite the achievement of clinical remission. In this setting, MRD reflects a low cellular burden of disease that remains undetectable by conventional morphological assessment and is typically evaluated using highly sensitive techniques such as multiparametric flow cytometry, PCR-based assays, or NGS.

In solid tumors, the concept of residual disease has been adapted to reflect fundamental biological and methodological differences. Residual disease is usually assessed from the detection of tumor-derived molecular signals, most commonly ctDNA that remains in the bloodstream following curative-intent interventions. Accordingly, the term Molecular Residual Disease (MoRD) is preferred, as it emphasizes that residual disease is defined by molecular detection. This terminology is consistent with the lexicon adopted by regulatory and scientific bodies, including the FDA, ESMO, BLOODPAC [42,43].

A concept complementary to MoRD is the Cellular Residual Disease (CRD), which describes the persistence of viable tumor cells, such as CTCs in blood or DTCs in organs like bone marrow. These cells are biologically active and have the potential to remain dormant, survive therapy, and eventually drive metastatic recurrence [44].

Importantly, MRD and CRD may not always co-occur: some patients may harbor CTCs without detectable ctDNA, or vice versa. Thus, integrating both approaches can enhance the sensitivity and accuracy of relapse risk stratification.

Liquid Biopsy in the neoadjuvant setting: In this clinical scenario, LB is intended for the detection of ctDNA while the primary tumor remains in situ and encompasses two temporally distinct but conceptually related assessments. Pre-neoadjuvant LB (Pre-NA-LB) corresponds to baseline ctDNA quantification prior to initiation of neoadjuvant therapy and serves as a reference for longitudinal monitoring of treatment response. A liquid biopsy sample collected after completion of neoadjuvant therapy but before surgical resection is referred to in the literature as a post-neoadjuvant or pre-operative LB; within ISLB, we recommend adopting the former term and the acronym Post-NA-LB. Assessment of ctDNA dynamics in Post-NA-LB samples can reveal response to neoadjuvant strategies through ctDNA clearance or, conversely, indicate incomplete response to therapy and/or the presence of residual viable tumor tissue. This information could assist in guiding subsequent surgical or adjuvant treatment decisions [45], [46].

Liquid Biopsy in the post-operative setting: In the post-operative scenario, MoRD is assessed after complete surgical resection of the primary tumor, with or without subsequent adjuvant therapy. In this context, MoRD refers to the detection of ctDNA in the absence of macroscopic disease. Unlike the neoadjuvant setting, in which the primary tumor remains in situ, post-operative MoRD positivity is interpreted as evidence of occult residual disease or micrometastatic dissemination persisting beyond surgical resection. MoRD positivity has been consistently associated with an increased risk of disease recurrence and represents a powerful prognostic biomarker. Accordingly, assessment of MoRD provides a non-invasive strategy for identifying patients at high risk of relapse and may support adjuvant treatment escalation or de-escalation strategies [47,48].

Currently, most of the developed MoRD assays focus in ctDNA detection. There are two main methodological approaches to ctDNA MoRD detection: i) tumor informed assays, which require prior sequencing of the patient's tumor tissue to design patient-specific tracking mutations and ii) tumor agnostic or plasma informed assays, which are based on fixed panels targeting common tumor-related genes and do not require matched tissue. Tumor-informed methods typically offer higher sensitivity but are more time and resource intensive. In addition, they require an adequate tumor specimen, which may limit their applicability [49]. By contrast, plasma-informed assays provide faster turnaround times and broader applicability, particularly when tumor tissue is unavailable or insufficient [42,44]. In the later scenario, TF measurement is crucial to ensure detection of tumor-related alterations.

###### 1.3.2.3.1 Landmark and longitudinal surveillance approaches

Two major strategies are used in MoRD evaluation: the landmark approach and longitudinal surveillance [50]. Although both rely on the use of LB after curative-intent therapy, they differ substantially in timing, sensitivity, and clinical implications.

The landmark approach refers to LB testing at a single predefined MoRD timepoint after completion of curative-intent therapy, typically 2–8 weeks after surgery, or at a fixed interval after completion of adjuvant therapy. The primary objective of this strategy is early post-treatment risk stratification to support treatment decision-making.

Longitudinal surveillance consists of repeated LB sampling at regular intervals during post-treatment follow-up. At present, this approach remains primarily investigational, and results from ongoing clinical trials are expected to clarify its clinical applicability, optimal sampling frequency and comparative sensitivity relative to landmark-based strategies.

##### 1.3.2.4 Liquid biopsy in advanced/metastatic stages of cancer

In advanced-stage cancers, LB plays a critical role throughout the patient's clinical course, providing real-time molecular information.

At the time of diagnosis, LB is increasingly used to characterize the tumor's molecular profile when tissue is unavailable, insufficient, inaccessible, or when rapid results are required. It also serves as a complementary source of molecular information, helping to capture heterogeneity across metastatic sites and enabling more accurate selection of targeted therapies or immunotherapy regimens.

During systemic therapy, serial LB monitoring enables dynamic assessment of treatment response and earlier identification of disease progression. This approach remains investigational and aims to provide opportunities for early therapeutic adaptation; however, its clinical applicability requires further investigated.

At the time of disease progression, LB is a key tool to elucidate mechanisms of resistance (MoR) and guide the selection of subsequent lines of therapy. MoR for TKIs are generally attributable to either on-target alterations or off-target (bypass) pathway activation, both of which can be effectively interrogated using NGS-based LB panels. By contrast, the assessment of histological transformation as a MoR remains more challenging. Nevertheless, emerging LB approaches incorporating multi-omic analyses, are increasingly providing informative signals suggestive of histological transformation and tumor lineage plasticity.

##### 1.3.2.5 Clonal hematopoiesis

Clonal hematopoiesis (CH) refers to an age-related expansion of blood cells descending from a single hematopoietic stem cell or progenitor cell, harboring specific, disruptive, and recurrent genetic variants. CH can lead to a clonal outgrowth in the absence of overt hematologic malignancies [51]. Although CH is not classified as a malignancy, it is increasingly recognized as a risk factor for several pathological conditions, including hematologic cancers, cardiovascular diseases, and other inflammatory and age-related disorders due to its association with altered immune response and systemic inflammation​. CH affects more than 10% of individuals over the age of 60 [52] and poses significant challenges for LB interpretation, particularly, in distinguishing tumor-derived mutations from age-related clonal variants in cfDNA analysis. However, emerging DNA-based approaches, including methylomic and fragmentomic profiling, have demonstrated high sensitivity and specificity in differentiating CH-associated alterations from tumor-derived somatic variants [53].

## 2 Conclusions

LB has matured into a versatile tool with roles spanning early detection, response assessment, MoRD monitoring, and real-time therapeutic guidance. As technologies grow more sensitive and the range of analytes and clinical contexts expands, standardized definitions become essential to ensure accurate interpretation, comparability of studies, and appropriate clinical use.

This document establishes a clear and harmonized terminology to support the scientific, clinical, and technological advancement of LB. By defining key analytes, workflows, biospecimens, and clinical applications, ISLB provides a unified reference that strengthens communication across disciplines and improves the consistency of research and diagnostic practice.

The framework presented here lays the foundation for future consensus efforts and will be periodically updated to reflect scientific progress. Through this evolving lexicon, ISLB reaffirms its commitment to guiding the field toward coherent, evidence-based integration of LB into precision medicine.

## ERB

N/A.

## Declaration of generative AI and AI-assisted technologies in the manuscript preparation process

During the preparation of this work the authors used ChatGPT and Perplexity in order to improve language clarity and readability (e.g., grammar, style). After using this tools the authors reviewed and edited the content as needed and take full responsibility for the content of the published article.

## Funding support

This work was co-funded by the European Union's EU4Health Programme under Grant Agreement No. 101232874 (SPARC project). Views and opinions expressed are however those of the author(s) only and do not necessarily reflect those of the European Union or HaDEA. Neither the European Union nor the granting authority can be held responsible for them.

## Declaration of competing interest

The authors declare the following financial interests/personal relationships which may be considered as potential competing interests:

Given their role in the editorial board of Journal of Liquid Biopsy, UM, EJL, MJS, NF, DG, OK, NL LR and CR declare no involvement in the peer review of this article and has no access to information regarding its peer review.

Authors declare that they have no known competing financial interests or personal relationships that could have appeared to influence the work reported in this paper.
